# A comprehensive dosimetric evaluation of using RapidArc volumetric‐modulated arc therapy for the treatment of early‐stage nasopharyngeal carcinoma

**DOI:** 10.1120/jacmp.v13i6.3887

**Published:** 2012-11-08

**Authors:** Monica W.K. Kan, Wicger Wong, Lucullus H.T. Leung, Peter K.N. Yu, Ronald W.K. So, Ashley C.K. Cheng

**Affiliations:** ^1^ Department of Oncology Princess Margaret Hospital Hong Kong SAR; ^2^ Department of Physics and Materials Science City University of Hong Kong Hong Kong SAR

**Keywords:** RapidArc, triple‐arc, peripheral doses, sliding window IMRT, early‐stage nasopharyngeal carcinoma

## Abstract

The purpose of this study was to investigate the potential benefits of using triple‐arc volumetric‐intensity modulated arc radiotherapy (RapidArc (RA)) for the treatment of early‐stage nasopharyngeal carcinoma (NPC). A comprehensive evaluation was performed including plan quality, integral doses, and peripheral doses. Twenty cases of stage I or II NPC were selected for this study. Nine‐field sliding window IMRT, double‐arc, and triple‐arc RA treatment plans were compared with respect to target coverage, dose conformity, critical organ sparing, and integral doses. Measurement of peripheral doses was performed using thermoluminescent dosimeters in an anthropomorphic phantom. While similar conformity and target coverage were achieved by the three types of plans, triple‐arc RA produced better sparing of parotid glands and spinal cord than double‐arc RA or IMRT. Double‐arc RA plans produced slightly inferior parotid sparing and dose homogeneity than the other two delivery methods. The monitor units (MU) required for triple‐arc were about 50% less than those of IMRT plans, while there was no significant difference in the required MUs between triple‐arc and double‐arc RA plans. The peripheral dose in triple‐arc RA was found to be 50% less compared to IMRT near abdominal and pelvic region. Triple‐arc RA improves both the plan quality and treatment efficiency compared with IMRT for the treatment of early stage NPC. It has become the preferred choice of treatment delivery method for early stage NPC at our center.

PACS numbers: 87.53.Bn, 87.55.D, 87.55.de, 87.55.dk, 87.56.ng

## I. INTRODUCTION

As a result of the technological advances of recent years, the use of highly conformal radiotherapy has become the norm used in the treatment of nasopharyngeal carcinoma (NPC). Recently, volumetric‐modulated arc radiotherapy (VMAT) has been introduced for clinical use in different treatment sites as a competing radiotherapy delivery modality to conventional intensity‐modulated radiotherapy with static gantry (IMRT).^1^, ^8^ VMAT is a novel extension of conventional IMRT, in which an optimized three‐dimensional (3D) dose distribution may be delivered in one or several gantry rotations, depending on the plan complexity. This concept has been clinically implemented in the Eclipse treatment planning software under the name RapidArc (RA). Conventional IMRT delivers fully intensity‐modulated radiotherapy fields with a multileaf collimator (MLC) from a finite number of fixed gantry angles, while RA delivers radiotherapy with MLC that changes the shape of the treatment field dynamically while the gantry rotates around the patient.^(9)^

Comparison between VMAT dose distribution in head and neck cancer and those obtained by conventional IMRT techniques have been reported by several investigators.^2^, ^8^ The head and neck cases chosen by previous investigators mostly involved a mixture of various sites including NP region, oropharynx, larynx, and hypopharynx. The target volumes of NPC are usually adjacent to a relatively larger volume of critical organs including brain stem, pituitary, temporal lobes, parotid glands, and optical structures. Therefore, assessment of the capabilities of different planning techniques for NPC cases should be separated from other head and neck sites. Lee et al.^(7)^ have done a pioneer investigation comparing between SmartArc‐based VMAT and seven‐field step‐and‐shoot IMRT for NPC. They concluded that dual‐arc VMAT produced similar target coverage and organ sparing as seven‐field step‐and‐shoot IMRT with faster delivery time. RapidArc using a different optimization method and delivery system from SmartArc used with Elekta treatment machine might lead to different results. Cheung et al.^(8)^ did a preliminary investigation on the use of RapidArc for three different NPC patients showing that RA produced reasonable plan quality for clinical use. Extensive comparison among double‐arc, triple‐arc RapidArc, and sliding window IMRT for NPC cases has not been reported. NPC cases with different staging involve different levels of complexity. Taking into account both early and advanced stage diseases together in the same evaluation might produce mixed results. Our current study compared both plan quality and treatment efficiency among double‐arc, triple‐arc RapidArc, and nine‐field sliding window IMRT, focusing on early stage NPC.

The use of different types of intensity‐modulated treatment techniques would deliver different amount of low doses to nontumor volumes, which may affect the probability of radiation‐induced second cancers. Comparison of peripheral doses between RA and sliding window IMRT has not been reported. Our study investigated both nontumor integral doses and peripheral doses leading to a more comprehensive evaluation of the treatment techniques. The total and nontumor integral doses for double‐arc, triple‐arc RA, and sliding window IMRT were estimated from the CT data and dose data retrieved from the treatment plans. The peripheral doses at different distances from the inferior field edges were measured using thermoluminescent dosimeters (TLDs) in an anthropomorphic phantom for comparison.

## II. MATERIALS AND METHODS

### A. Patient selection and contouring

Twenty patients with early stage NPC were chosen for this study. Thirteen patients who were previously treated with nine‐field sliding window IMRT were replanned with double‐ and triple‐arc RA technique using the Eclipse version 8.6 (Varian Medical Systems Inc., Palo Alto, CA), while the other seven patients were treated with triple‐arc RA and were then replanned with double‐arc RA and IMRT for comparison purposes.

The primary gross tumor volume $\left({\mathrm{GTV}}_{P}\right)$ covered the gross tumor plus the entire nasopharynx down to caudal border of C1 vertebra. The nodal gross tumor volume $\left({\mathrm{GTV}}_{N}\right)$ encompassed any positive lymph nodes as defined by a short axis of greater than 1 cm, the presence of necrotic center, or extracapsular infiltration. The boost clinical target volume $\left({\mathrm{CTV}}_{B}\right)$ and the nodal clinical target volume $\left({\mathrm{CTV}}_{N}\right)$ were formed by adding 0.5 cm margin to ${\text{GTV}}_{P}$ and ${\text{GTV}}_{N}$, respectively (0.1–0.2 cm margin if abutting neurological structures or bone). The high risk clinical target volume $\left({\mathrm{CTV}}_{H}\right)$ was created by adding 1 cm margin to ${\text{CTV}}_{B}$ and ${\text{CTV}}_{N}$, plus minimal coverage for high‐risk elective sites including lower half of sphenoid sinus, anterior half of clivus, base of skull, petrous tips, posterior third of nasal cavity and maxillary sinuses, pterygoid fossae, parapharyngeal spaces, and lymph nodes bilaterally (posterior part of level Ib, level II to III and retropharyngeal). The low risk clinical target volume $\left({\mathrm{CTV}}_{L}\right)$ covered bilateral level IV and Vb nodal regions unless positive lymph nodes were present where ${\text{CTV}}_{H}$ would extend into level IV and Vb to 0.15 cm below caudal border of ${\text{CTV}}_{N}$. The ${\text{PTV}}_{B}$, ${\text{PTV}}_{N}$, ${\text{PTV}}_{H}$, and ${\text{PTV}}_{L}$ were produced by adding 0.3 cm to ${\text{CTV}}_{B}$, ${\text{CTV}}_{N}$, ${\text{CTV}}_{H}$, and ${\text{CTV}}_{L}$, respectively. Seventy (70) Gy was prescribed to the ${\text{PTV}}_{B}$ and ${\text{PTV}}_{N}$, 60 Gy was prescribed to the ${\text{PTV}}_{H}$, and 54 Gy was prescribed to the ${\text{PTV}}_{L}$. The prescription dose was given in 35 daily fractions. The goal was to give at least 95% of the PTV and 100% of the GTV the prescribed dose, and no more than 5% of the PTV would receive 107% of the prescribed dose. The dose constraints to organs at risk are summarized in Table 1.

**Table 1:** The dose constraints to organs at risk.

*Organs at Risk*	*Maximum*	*Dose Volume Constraints*
Brain stem	54 Gy	
Brain stem + 3 mm		Less than 1% should receive up to 60 Gy
Spinal cord	45 Gy	
Spinal cord + 5 mm		Less than 1% should receive up to 50 Gy
Optic nerves/chiasm	54 Gy	Less than 1% should receive up to 60 Gy
Temporal lobes	60 Gy	Less than 1% should receive up to 65 Gy
Parotid glands		mean dose ≤26 Gy or <50% to exceed 30 Gy
Eyes	45 Gy	Mean dose <35 Gy
Ears	58 Gy	Mean dose <50 Gy
Lens	6 Gy	
Pituitary	45 Gy	Less than 1% should receive up to 65 Gy
Brachial plexus	63 Gy	

### B. IMRT planning

The IMRT plans were created with sliding window technique using nine evenly distributed coplanar beam directions. All beam energies were 6 MV and modulated with 120 multileaf collimator from a linear accelerator (Clinac 6EX, Varian Medical Systems). Optimizations and dose calculations were done with Eclipse version 8.6.15. The optimization dose priorities were similar for all cases. Giving enough dose coverage to PTVs and limiting the maximum doses to brain stem, spinal cord, and optic nerves were given the highest priority, followed by reducing the dose to parotid glands. Lower priorities were given to the other critical organs. An optimization criterion was also assigned to an organ representing normal tissues, which was defined as the body volume in the CT dataset minus the PTV leaving 3 mm gap. Dose calculation was performed with AAA using a calculation grid of 2.5 mm. Both cold and hot areas in the PTVs were reduced to minimal by delineating them as virtual organs for further optimization. When reoptimization became ineffective, fluence editing was used routinely for our IMRT plans to minimize hot and cold areas. Editing fluence map for each beam direction is allowed by the Varian Eclipse planning software. It was proved by a previous study that manually editing IMRT fluence maps effectively controls hot and cold areas that optimization cannot control.^(10)^ This method has been routinely used in all our IMRT treatment planning. All the IMRT planning in the current study were generated by several well‐experienced expert planners who were able to generate plans with similar high quality.

### C. RA planning

RA was implemented as described in a publication by Otto.^(11)^ Optimizations and dose calculations were performed with the same treatment planning system as used in IMRT planning (Eclipse version 8.6.15). It allows for the use of multiple arc fields, nonzero couch angles, and avoidance sectors. A plan can be created using a maximum of 10 arc fields with a maximum total arc length of 1000°. The optimization method was discussed in detail by Oliver et al.^12^, ^13^ The final dose calculation was performed in Eclipse by the same version of AAA algorithm as the IMRT plans, with a grid resolution of 2.5 mm.

The limit of travel for the MLC leaf extends from the carriage was 14.5 cm. In this study, the collimator opening along the leaf travel direction was confined to about 14.5 cm or slightly more, so that each leaf could move as freely as possible across the whole field for optimal modulation. Several preliminary tests done by the authors showed that this could reduce hot areas inside the target volumes. The authors chose to use both double and triple arcs for each RA planning. The double‐arc RA consists of two complete arcs, each with 358° arc length, while the triple‐arc RA plans were created using two complete arcs plus one partial arc (moving from gantry angle of 240° to 120° in the clockwise direction). The couch angles of all arcs were zero degrees.

Nonzero collimator angles were used for all RA plans to minimize the tongue‐and‐groove effect. The optimization aims were the same as those for sliding window IMRT plans. However, the optimization template used in RA planning was different from that of IMRT due to the differences in optimization algorithm between the two techniques. During the optimization process, both IMRT and RA used the same approach for dose estimation (the multiresolution dose calculation), but different approaches for plan optimization. A fluence‐based optimization approach was used for IMRT, for which the dose‐volume optimizer (DVO) was used to generate the optimal fluence map for each field. A direct aperture optimization approach was used for RA, for which the progressive resolution optimizer (PRO) iteratively changed the dynamic delivery variables such as MLC leaf positions, dose rate, and gantry rotational speed during optimization using a set of penalty functions.^(11)^ It was observed from our initial RA planning experience that using the same template settings as in IMRT planning would lead to suboptimal plans.

The same initial optimization template was used in double‐arc and triple‐arc RA plans for all patients. About four to five reoptimizations were repeated to improve the conformity and dose homogeneity, depending on the complexity of the plan. All the RA plans were generated by one single planner to minimize variation in plan quality due to the dependence on operator skill.

### D. Plan comparison

Plan comparisons were performed for all the 20 early‐stage NPC cases among double‐arc RA, triple‐arc RA, and nine‐field IMRT plans. The parameters used for plan comparisons include PTV dose homogeneity, conformity, critical organ sparing, and the number of monitor units (MU) required. The plan conformity was evaluated using the conformation number, CN, which was defined as the product of $V_{T,\text{ref}}/\text{VT}$ and $V_{T,\text{ref}}/V_{\text{ref}}$, where $V_{T,\mathrm{ref}}$ represents the volume of the target receiving a dose equal to or greater than the reference dose, VT represents the physical volume of the target, and $V_{\text{ref}}$ represents the total tissue volume receiving a dose equal to or greater than the reference dose.^(14)^ The reference dose used to compute the CN is the prescription dose. The dose homogeneity index (HI) described by Wu et al.,^(15)^ was used to describe the dose homogeneity of the target volumes, which was defined as:

(1) $$HI=\frac{D_{1\%}-D_{99\%}}{D_{p}}\times 100$$

where *HI* is the homogeneity index, while $D_{1\%}$ and $D_{99\%}$ are the doses delivered to 1% and 99% of the PTV, and $D_{p}$ represents the prescription dose. A lower HI value indicates a more homogeneous target dose. Besides, for all the plans, $V_{<95\%}$ (the volume receiving less than 95% of the reference dose), $V_{<100\%}$ and $V_{>105\%}$ were scored for PTVs. For the organ at risk (OAR), the mean dose $\left(D_{\mathrm{mean}}\right)$ to the parotid glands, the volume of parotid glands receiving more than 30 Gy ($V_{30\text{Gy}}$), the dose encompassing 1% ($D_{1\%}$) of the volume for brain stem, the planning organ at risk volume (PRV) brain stem (brain stem with 3 mm margin), spinal cord, PRV spinal cord (spinal cord with 5 mm margin), optic chiasm, optic nerve and brachial plexus, and the mean doses to lens, eyes, inner ears, and pituitary were also reported. The Wilcoxon matched‐pair signed rank test was used to compare the results between the couples, IMRT against triple‐arc RA plans, and double‐arc against triple‐arc RA plans. The threshold for statistical significance was p≤0.05. All statistical tests were two‐sided, and all analyses were performed using the Statistical Package for Social Sciences, version 11.0 (SPSS, Chicago, IL).

### E. Comparison of delivery time

The delivery time was defined as the time span from the first beam‐on to the last beam‐off. For most of the RA and IMRT plans, the delivery time of the corresponding quality assurance plans was retrieved directly from the treatment history of the Varian system, where the beam‐on and beam‐off time of each arc or field was recorded properly. Since some plans were not delivered at all, the delivery time was estimated by a simple mathematical model, taking into account the gantry/collimator movement and data transfer time.

For IMRT plans:

(2) $$Estimated\;delivery\;time=\sum_{i=1}^{N}{BeamOnTime}_{i}+T_{g}(N_{g}-1)+T_{s}(N_{s})$$

where *N* is total number of beams of the corresponding plan, $T_{g}$ is the average time for gantry movement between each field, $N_{g}$ is the number of gantry angles, $T_{s}$ is the average setup time for split field, $N_{s}$ is the number of split fields. The limit of travel for the MLC leaf extending from the carriage was 14.5 cm. Split fields were required for large PTV. The *BeamOnTime* was the irradiation time for each field calculated by Eclipse based on the default dose rate when the plan was approved. For RA plans:

(3) $$Estimated\;Treatment\;Time=\sum_{i=1}^{N}{BeamOnTime}_{i}+T(N-1)$$

where *N* is total number of arcs, and *T* is the average setup time in‐between arcs, including the time for collimator rotation.

The average setup times for $T_{g}$, $T_{s}$, and T were obtained from previous treatment history of 10 patients, and were 0.43±0.05  min, 0.36±0.06  min, and 0.57±0.06  min, respectively.

### F. Integral doses

The integral dose of an organ is reported as the sum of all dose voxels times their mass. It is given by:

(4) $$Integral\;dose=\sum_{i=1}^{N}D_{i}\cdot m_{i}=\sum_{i=1}^{N}D_{i}\cdot v_{i}\cdot \rho_{i}$$

where *N* is the number of voxels, $D_{i}$ is the dose to the voxel, $m_{i}$ is the mass of the voxel, and $v_{i}$ and $\rho_{i}$ are the volume and density of voxel i in the organ.^(12)^ All integral doses were computed with heterogeneous tissue density correction by retrieving the CT and dose data from each voxel of the treatment plan. Comparison was made based on data from 10 patients for the double‐arc, triple‐arc RA, and IMRT plans. Both total and nontumor integral doses (NTID) were estimated. NTID is the integral dose to all OARs and tissue together excluding the PTVs. To compare the NTID for different doses per fraction, the biological effective dose (BED) was calculated using the following equation:

(5) $$BED=nd\left(\frac{1+\frac{d}{\overset{\alpha }{\;}/\underset{\beta }{\;}}}{1+\frac{d_{ref}}{\alpha /\beta }}\right)$$

where $d_{\text{ref}}$ was selected as 2 Gy, *n* is the number of fractions, *d* is dose per fractions, and α/β was selected as 3 for OAR and 10 for target. The unit of BED is ${\text{Gy}}_{2}$, where 2 represents the reference dose (2 Gy) per fraction.^(16)^

### G. Measurement of peripheral doses

**Figure 1:**
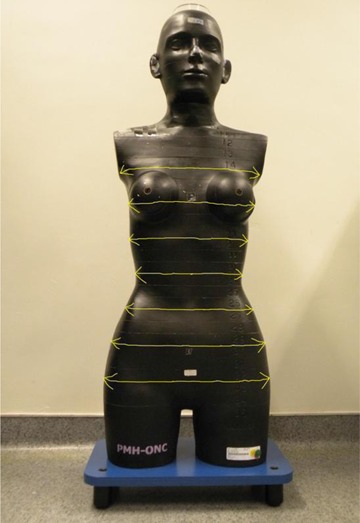
The anthropomorphic phantom for measurement of peripheral doses using TLDs. TLDs were placed symmetrically about the central axis at levels indicated by the yellow lines.

The peripheral doses for both triple‐arc RA and sliding window IMRT plans of 10 patients were measured using an anthropomorphic phantom (the RANDO phantom, The Phantom Laboratory, Salem, NY), as shown in Fig. 1. The phantom includes bone, lung, and soft tissue compositions formulated for accurate simulation for therapy energies. The whole phantom was divided into 26 sections, each of 25 mm thickness. TLDs were located about 10 cm below the inferior edge of the treatment fields, and then every 7.5 cm towards the inferior direction down to the level of the pelvis. Four TLDs were placed symmetrically about the central axis on every selected slice. The isocenter position was chosen to be near the actual isocenter of each patient plan. TLD 700H (LiF:Mg,Cu,P) pellets measuring 3.6 mm diameter by 0.38 mm thick were chosen for this experiment. They are of high sensitivity, minimal photon energy dependence and exhibit a lack of supralinearity which enables accurate measurements in low‐dose regions.^(17)^ The Harshaw Model 5500 TLD reader (Thermo Fisher Scientific Inc., Waltham, MA) was used to provide a precise control of the heating regime. All TLDs were annealed at 245°C for 15 min and then 100°C for 2 hrs before irradiation. To remove the short half‐life peaks, the irradiated TLDs were preheated at 145°C for 10 sec. Data was acquired at 260°C for 23.3 sec. The whole set of TLDs was irradiated with a known dose using 10×10 cm 6 MV X‐ray beam. Each TLD chip was assigned a sensitivity value that related its individual dose response to the mean dose response of the set. The sensitivity value was taken as the average of three irradiations. Assigning a sensitivity correction factor to each TLD chip improved the accuracy of our measurement.

## III. RESULTS

### A. Comparison between RA and IMRT

Table 2 summarizes the results of conformity, dose homogeneity, PTV coverage, and doses to OARs averaged over 20 patients. The average CN values for PTVs reflect similar conformity among the three types of planning techniques. When looking at the $V_{<95\%}$, there is no significant difference in PTV coverage. The highest averaged HI value for ${\text{PTV}}_{B}$ or ${\text{PTV}}_{B+N}$ of the double‐arc plans indicated that it produced slightly inferior dose homogeneity than that of the IMRT and triple‐arc RA plans. $V_{>105\%}$ was 0.4% for IMRT, 5.6% for triple‐arc RA, and 7.8% for the double‐arc RA, indicating that more hot areas appeared in RA plans.

**Table 2:** Plan comparison among nine‐field IMRT, double‐arc RA, and triple‐arc RA plans (average over 20 patients).

*Parameter*	*Nine‐field IMRT*	*Double‐arc RA*	*Triple‐arc RA*	p^a^ *(IMRT vs. Triple RA)*	p^a^ *(Double RA vs. Triple RA)*
CN(${\text{PTV}}_{B}$/${\text{PTV}}_{B+N}$)	0.86±0.02	0.85±0.03	0.86±0.02	0.63	0.10
CN(${\text{PTV}}_{H}$)	0.86±0.02	0.87±0.02	0.87±0.03	0.20	0.26
HI (${\text{PTV}}_{B}$/${\text{PTV}}_{B+N}$)	5.14±0.41	5.92±0.38	5.30±0.57	0.43	0.00
$V_{<95\%}$(${\text{PTV}}_{B}$/${\text{PTV}}_{B+N}$), %	0.01±0.02	0.00±0.00	0.00±0.00	0.11	0.16
$V_{<95\%}$(${\text{PTV}}_{H}$), %	0.08±0.09	0.10±0.10	0.13±0.12	0.10	0.16
$V_{<100\%}$(${\text{PTV}}_{B}$/${\text{PTV}}_{B+N}$), %	2.64±1.22	1.61±0.68	1.23±0.55	0.00	0.02
$V_{<100\%}$(${\text{PTV}}_{H}$), %	1.31±0.81	1.62±0.65	1.52±0.62	0.25	0.81
$V_{<105\%}$(${\text{PTV}}_{B}$/${\text{PTV}}_{B+N}$), %	0.35±0.91	7.84±3.55	5.58±2.02	0.00	0.00
$D_{1\%}$(Brain stem), Gy	49.56±1.51	50.35±1.20	49.12±1.27	0.23	0.00
$D_{1\%}$(PRV brain stem), Gy	55.04±1.48	54.38±0.99	53.56±1.20	0.00	0.00
$D_{1\%}$(Spinal cord), Gy	41.44±1.63	41.28±1.18	40.16±0.84	0.00	0.03
$D_{1\%}$(PRV spinal cord), Gy	48.45±1.40	46.31±1.81	45.73±1.49	0.00	0.05
$D_{1\%}$ (Optic Chiasm), Gy	16.23±9.21	14.98±7.90	14.90±7.90	0.53	0.72
$D_{1\%}$ (Lt Optic Nerve), Gy	17.75±10.83	15.60±9.70	16.65±9.18	0.63	0.06
$D_{1\%}$(Rt Optic Nerve), Gy	19.86±12.96	16.64±10.62	16.40±10.80	0.07	0.35
$D_{1\%}$(Brachial plexus), Gy	60.58±1.61	60.70±1.32	60.49±1.45	0.72	0.30
$D_{\text{mean}}$(Lt parotid), Gy	30.02±3.27	31.60±3.54	26.71±2.53	0.00	0.00
$D_{\text{mean}}$ (Rt parotid), Gy	30.75±4.82	32.06±4.44	27.93±4.28	0.00	0.00
$V_{30\text{Gy}}$ (Lt parotid), %	38.18±8.69	44.11±10.89	30.98±6.13	0.00	0.00
$V_{30\text{Gy}}$ (Rt parotid), %	38.89±11.33	44.72±11.57	32.99±9.67	0.00	0.00
$D_{\text{mean}}$ (Lt lens)	3.82±0.42	4.30±0.54	4.30±0.33	0.00	0.91
$D_{\text{mean}}$ (Rt lens)	3.92±0.51	4.11±0.51	4.23±0.48	0.01	0.41
$D_{\text{mean}}$ (Lt eye)	5.40±1.19	6.04±1.80	6.64±2.94	0.14	0.84
$D_{\text{mean}}$ (Rt eye)	6.06±1.64	5.66±1.45	6.23±1.95	0.08	0.12
$D_{\text{mean}}$ (Pituitary)	38.26±10.94	36.11±9.26	36.61±9.26	0.13	0.88
$D_{\text{mean}}$ (Lt ear)	48.78±4.05	46.29±2.87	47.47±3.39	0.09	0.28
$D_{\text{mean}}$ (Rt ear)	48.00±3.05	45.85±3.05	45.99±5.06	0.02	0.94

a Statistical significance (p<0.05) is reported between couples, IMRT vs. triple RA, and double RA vs. triple RA, from Wilcoxon signed rank test.

When compared with IMRT plans, the mean doses to parotids were reduced by 12% and $V_{30\text{Gy}}$ was reduced by 17% by using triple‐arc RA. The average mean doses to parotids $\left(D_{\mathrm{mean}}\right)$ were comparable between IMRT plans and double‐arc RA, while the average $V_{30\text{Gy}}$ of double‐arc RA was 15% higher than that of IMRT, indicating that double‐arc RA plans actually produce inferior sparing of the parotid glands than IMRT. The comparisons of dose distribution in (Fig. 2a) and dose volume histograms (DVHs) in (Fig. 2b) show the improved parotid sparing for the triple‐arc RA vs. IMRT for a typical patient.

**Figure 2(a):**
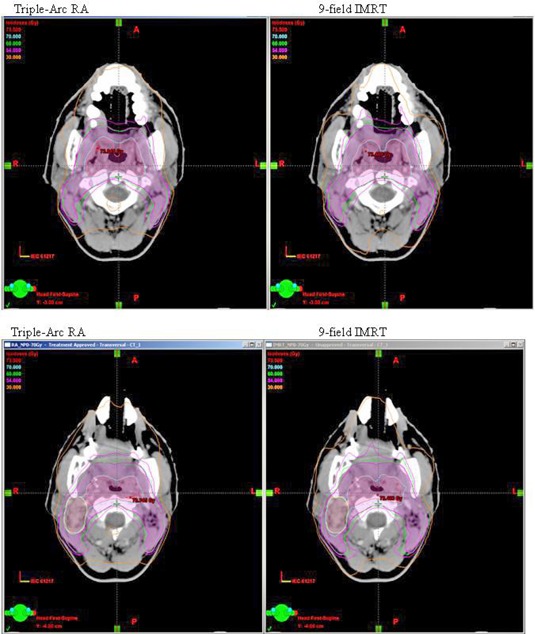
Comparison of dose distribution in the axial planes between triple‐arc RA vs. IMRT for a typical early stage NPC patient.

The triple‐arc RA plans produced slightly better sparing of the spinal cord compared to both IMRT and double‐arc RA plans. The average $D_{1\%}$ to the spinal cord was 41.4 Gy, 41.3 Gy, and 40.2 Gy for the IMRT, double‐arc, and triple‐arc RA plans, respectively. It can be observed that the $D_{1\%}$ to the PRV brain stem and PRV spinal cord were reduced by 1.5 Gy and 2.8 Gy, respectively, by using triple‐arc RA plans compared with IMRT. There was slightly less improvement of sparing to these organs by using double‐arc RA plans. The sparing of other sensitive serial structures was not significantly different among the three types of plans.

The average number of MUs for the nine‐field IMRT plans was 1539 ± 202, while that for the double‐arc and triple‐arc RA plans was 678 ± 107 and 706 ± 92, respectively. The use of RA plans led to a significant reduction of MUs (more than half), while the difference in required MUs between double‐arc RA and triple‐arc RA plans was statistically insignificant (p=0.313). The delivery time including the beam‐on time and the time for gantry and collimator movement of each plan was also recorded. The averaged value was 8.8±1.0 , 3.0±0.1 , and 4.5±0.2  minutes for IMRT and double‐arc and triple‐arc RA plans, respectively.

**Figure 2(b):**
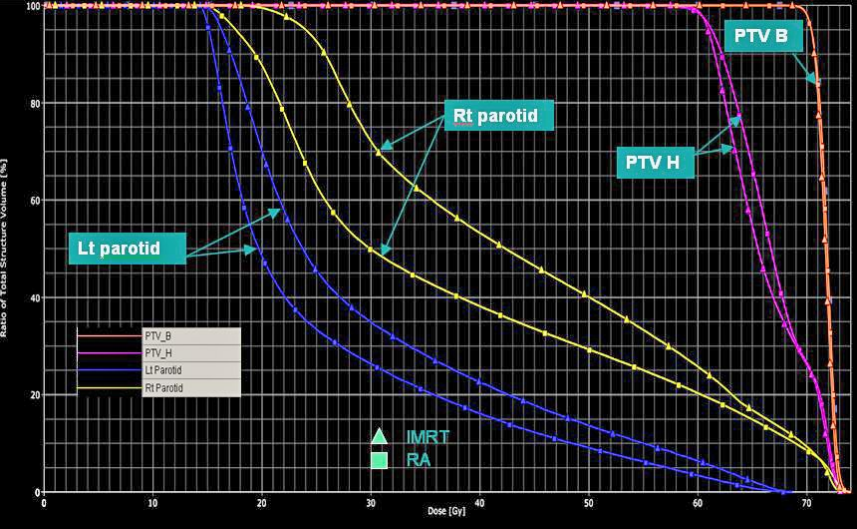
Dose volume histograms (DVHs) of PTVs and parotids between triple‐arc RA vs. IMRT for a typical early stage NPC patient.

### B. comparison of integral doses

The averaged total integral doses and NTID with and without BED corrections of 10 patients for the double‐arc, triple‐arc RA plans, and IMRT plans are listed in Table 3. When BED correction was not applied, the averaged total integral dose and NTID of double‐arc RA plans were only about 1% smaller than those of the triple‐arc RA plans. There was no statistical significant difference in both total integral dose and NTID with BED corrections among the three types of plans for early‐stage NPC cases.

**Table 3:** Summary of integral doses for nine‐field IMRT, double‐arc RA, and triple‐arc RA plans (average over 10 patients).

*Parameter*	*Nine‐field IMRT*	*Double‐arc RA*	*Triple‐arc RA*	*(IMRT vs. Triple RA)*	p^a^ *(Double RA vs. Triple RA)*
Total integral dose without BED correction, Gy·kg	207.0±28.8	207.3±30.0	208.8±29.9	0.29	0.05
Total integral dose with BED correction, ${\text{Gy}}_{2}\cdot \text{kg}$	176.6±24.8	176.0±25.1	177.5±25.2	0.58	0.08
NTID without BED correction, Gy·kg	156.3±21.7	155.9±23.1	157.5±23.3	0.45	0.04
NTID with BED correction, ${\text{Gy}}_{2}\cdot \text{kg}$	126.5±17.4	125.2±18.4	126.7±18.5	0.77	0.07

a Statistical significance (p<0.05) is reported between couples, IMRT vs. triple RA, and double RA vs. triple RA, from Wilcoxon signed rank test.

### C. Comparison of peripheral doses

The averaged peripheral doses for 10 patient plans at different distances inferior to the treatment field edges for both IMRT plans and triple‐arc RA plans are shown in Table 4. For each plan, peripheral doses were measured for a single fraction, and then multiplied by a total of 35 fractions, for comparison. At shorter distances below the field border, there is no significant difference in peripheral doses between RA and IMRT plans. At distances more than about 30 cm from the field edges, that is from the abdomen to pelvis, the averaged peripheral doses from the RA plans were almost half of that from the IMRT plans. At levels near the gonads, it was 4.2 cGy and 8.8 cGy for the whole treatment for the RA and IMRT plan, respectively.

**Table 4:** The averaged total peripheral doses in cGy over 35 fractions measured for 10 patient plans at different distances inferior to the treatment field edges for both IMRT plans and triple‐arc RA plans.

*Distances (from the inferior field edge in cm, position)*	*Nine‐field IMRT*	*Triple‐arc RA*
10.0 cm, near thorax and breast	162.4±28.4	144.6±56.9
17.5 cm, near thorax and breast	82.8±9.5	59.4±13.7
25.0 cm, upper abdomen	39.1±4.0	27.4±5.8
32.5 cm, abdomen	27.0±3.4	14.7±3.0
40.0 cm, lower abdomen	24.0±2.8	11.0±2.3
47.5 cm, pelvis	16.4±1.4	7.3±1.3
55.0 cm, pelvis	8.8±0.8	4.2±0.7

## IV. DISCUSSION

Some investigators showed that double‐arc VMAT could produce equivalent or better plan quality with more rapid treatment delivery for head and neck cases when compared with IMRT.^2^, ^3^, ^4^, ^6^ Other investigators found mixed results.^1^, ^8^ Sometimes, it is difficult to draw a definite single conclusion for all head and neck cases with different staging due to the differences in levels of target complexity. Our initial experience of using RA in some of the advanced stage NPC cases with very extensive target volumes did not produce very promising plan quality. On the other hand, the use of a different planning and delivery system might also lead to different results. Lee et al.^(7)^ found that slightly better sparing for the majority of the OARs except spinal cord and oral cavity by using SmartArc‐based dual‐arc VMAT compared to seven‐field step‐and‐shoot IMRT for NPC. Our investigation focused merely on early‐stage NPC proved that the use of triple‐arc RA produced comparable dose conformity and target dose homogeneity, as well as better sparing of the parotid glands, spinal cord, PRV spinal cord, and PRV brain stem compared with nine‐field sliding window IMRT. Although double‐arc RA produced similar conformity as IMRT, it did not produce any significant improvement in organ sparing. It actually resulted in slightly inferior sparing of parotid glands and target dose homogeneity. Compared with double‐arc RA plans, triple‐arc RA plans consist of more control points and a higher degree of freedom for possible leaf positions, leading to a higher degree of modulation. Reduction of doses to the spinal cord could be beneficial to NPC patients with recurrent or locally persistent diseases, especially when a second course treatment is necessary. Better sparing of parotid glands is important to early‐stage NPC patients to preserve a better quality of life. In addition, there is no statistically significant difference in the required MUs between double‐arc RA and triple‐arc RA. The use of double‐arc RA could only save about 1.5 minutes of delivery time compared to triple‐arc RA. Triple‐arc RA already saved 50% of MUs and half of the delivery time, as compared with IMRT. For early‐stage NPC cases, triple‐arc RA could be the best choice among the three modes of investigated treatment for both better plan quality and treatment efficiency. The only disadvantage of using triple‐arc RA is that the planning usually takes more time. The planning for IMRT and double‐arc RA usually takes about 1 day, while that for triple‐arc takes about one‐and‐a‐half days. The longer optimization and dose calculation times due to the use of three arcs led to slower planning cycles. For advanced‐stage NPC cases, a higher degree of modulation is required due to the involvement of a larger volume of PTVs and shorter distances between OARs and PTVs. The amount of improvement in organ sparing produced using triple‐arc RA as compared to IMRT observed in this work might not be reproduced for the advanced cases, and requires further investigation.

Regarding comparison of target dose homogeneity between VMAT and conventional IMRT, Guckenberger et al.^(2)^ reported that double‐arc SmartArc produced identical dose homogeneity, while triple‐arc resulted in improved dose homogeneity as compared to conventional IMRT for pharyngeal and laryngeal cases. The study of Verbakel et al.^(3)^ concluded that double‐arc RA produced better dose homogeneity than single‐arc RA and seven‐field sliding window IMRT for head and neck cases. Lee et al.^(7)^ concluded that dual‐arc SmartArc VMAT produced better target dose homogeneity than conventional seven‐field IMRT for NPC cases. Our results showed that RA plans produced more hot areas when compared with IMRT plans. During IMRT planning, cold and hot areas were contoured as virtual structures, and optimization criteria were given to reduce the amount of hot and cold areas until there was not much improvement by further optimization. However, most of the times, reoptimization only placed the hot and cold areas at different locations. At times when reoptimization became ineffective, the user would edit the fluence map to further improve the plan quality. The technique of fluence editing was our usual practice to reduce hot areas in IMRT planning. It could be noticed from our results that the average percentage volume of ${\text{PTV}}_{B}/{\text{PTV}}_{B+H}$ receiving a dose greater than 105% of the reference dose was only 0.4%. For RA plans, the technique of fluence editing could not be used in the planning software. This was because the dose optimization employed a direct aperture‐based method in which MLC leaf positions and weights were constrained such that the aperture shapes and MU values were physically achievable in practice. The only method to reduce hot and cold areas was by reoptimizing. As a result, there were relatively more hot areas in RA plans than in IMRT plans. From the judgment of our planning experience, it was believed that if the technique of fluence editing was not used in our conventional IMRT planning, the target dose homogeneity would be more comparable between triple‐arc RA and IMRT. However, it was only about 5% of ${\text{PTV}}_{B}/{\text{PTV}}_{B+H}$ receiving a dose greater than 105% of the reference dose using triple‐arc plans. This was considered clinically acceptable.

Some investigations were done comparing the total integral doses for helical tomotherapy, single‐arc, double‐arc VMAT, and IMRT using several different water equivalent virtual phantoms with artificial contours.^(14)^ Their results did not show any significant differences among IMRT, and single‐arc and double‐arc VMAT plans. Our estimation of nontumor integral doses has taken into account tissue inhomogeneity, patient geometry, and biological effective dose. The estimated nontumor integral doses for the three different modes of intensity‐modulated treatment techniques in our study also did not show any significant difference. It can be observed that the increase in arc angles in VMAT did not increase the nontumor integral dose.

It was observed from the measurement of peripheral doses that the reduction of doses associated to the use of RA increased as the out‐of‐field distances increased. The triple‐arc RA produced almost 50% reduction of peripheral doses at distances longer than 30 cm from the field edge near the pelvic region. Three of the major sources of peripheral doses include leakage from the treatment machine head, the scatter from secondary collimators and beam modifiers, and internal scatter originating in the patient. The amount of scatter from secondary collimators depends on the configuration of the machine head and the multileaf collimator. Both the RA and IMRT techniques used in this study were delivered with the same treatment machine with similar sizes of collimator and MLC openings. The patient integral doses of the two techniques estimated from the CT images were very close to each other. The difference in peripheral doses at a distance (10 cm) closer to the field edge between RA and IMRT was small. It was therefore believed that there was no significant difference in collimator and phantom scatter doses between the two techniques. The average number of MUs of the 10 IMRT plans and the 10 RA plans was 1432 and 594, respectively. On average, the number decreased by 58% when using RA plans instead of IMRT plans. The differences in peripheral doses should mainly be due to the reduction in radiation leakage from the machine head by using triple‐arc RA with reduced monitor units. The reduction of primary leakage dose by using RA decreases the total body exposure, and therefore might also decrease the risk of developing secondary cancers.^(18)^ Exposure of adult from radiotherapy in the abdominopelvic region may result in induction of genetic disorders in future generations.^(19)^ The significant reduction in peripheral doses near the pelvic region is of particular interest to the radiosensitive structures such as ovaries, testes, and fetus.

## V. CONCLUSIONS

Triple‐arc RA plans provide better sparing of parotid glands, spinal cord, PRV spinal cord, and PRV brain stem than both double‐arc RA and nine‐field sliding window IMRT plans for early‐stage NPC cases. Similar dose conformity and sparing of other organs were observed among the three delivery techniques. The number of MUs and delivery time were both reduced by approximately 50% when triple‐arc RA was employed, as compared to the sliding window IMRT. The use of double‐arc RA plans resulted in slightly inferior target dose homogeneity and parotid sparing, while its use could not save any monitor units compared with triple‐arc RA plans. Our investigations also showed that the use of triple‐arc RA plans did not change the nontumor integral doses within the regions of CT dataset. It reduced the peripheral doses by half at a distance longer than 30 cm near the lower abdomen and pelvis. Three arcs are the optimum number of arcs to be used in RA to gain both better plan quality and treatment efficiency for early‐stage NPC cases. It has become the choice of treatment delivery method for early‐stage NPC in our center. The use of RA for more advanced‐stage NPC cases requires further investigation.
